# Workplace Intervention for Reducing Sitting Time in Sedentary Workers: Protocol for a Pilot Study Using the Behavior Change Wheel

**DOI:** 10.3389/fpubh.2022.832374

**Published:** 2022-04-12

**Authors:** Samson O. Ojo, Daniel P. Bailey, Angel M. Chater, David J. Hewson

**Affiliations:** ^1^Institute for Health Research, University of Bedfordshire, Luton, United Kingdom; ^2^Quality Improvement, Northampton General Hospital NHS Trust, Northampton, United Kingdom; ^3^Institute for Sport and Physical Activity Research, Centre for Health, Wellbeing and Behaviour Change, University of Bedfordshire, Bedford, United Kingdom; ^4^Division of Sport, Health and Exercise Sciences, Department of Life Sciences, Brunel University London, Uxbridge, United Kingdom; ^5^Centre for Physical Activity in Health and Disease, Brunel University London, Uxbridge, United Kingdom; ^6^Centre for Behaviour Change, University College London, London, United Kingdom

**Keywords:** sedentary behavior, Behavior Change Wheel, intervention, desk-based employees, office workers, pilot study, protocol

## Abstract

The workplace is a major contributor to excessive sitting in office workers. There are a wide array of adverse effects of high volumes of sitting time, including an increased risk of type 2 diabetes and depression. Active workstations can be used in effective interventions to decrease workplace sitting. However, there are a lack of interventions that have been developed using a systematic process that is informed by participant needs and a framework for identifying the most appropriate content for the intervention. Applying these methods could increase adherence and potential effectiveness of the intervention. Therefore, the purpose of this pilot study is to examine the feasibility, acceptability, and efficacy of a tailored workplace intervention to reduce and break up sitting in office workers that has been developed using the Behavior Change Wheel and the APEASE (Acceptability, Practicability, Effectiveness/cost-effectiveness, Affordability, Safety/side-effects, Equity) criteria. This article reports the protocol for this study that is currently ongoing. Participants will be cluster-randomized (by offices) to control and intervention groups. The evaluation of the intervention includes determining feasibility by assessing participant recruitment, retention and data completion rates. Adherence to the intervention will be assessed based on daily sitting and standing time relative to guidelines provided to participants as part of the intervention. Outcome measures also include productivity measured using Ecological Momentary Assessment, absenteeism, presenteeism, cardiometabolic risk markers, and wellbeing. The findings of this study will inform the effective design and implementation of interventions for reducing and breaking up sitting in office workers.

## Introduction

Sedentary behavior has been described as any waking behavior in a sitting, reclining or lying posture that has an energy expenditure of ≤ 1.5 metabolic equivalents (METs) (1). In the United Kingdom, high levels of sedentary behavior have been reported with male and female employees sitting more than 11 h/day on average (2). Studies have identified the workplace as a major contributor to excessive sitting (3) with one study reporting that office workers spent 79% of their working day sitting and 42% of their work hours engaging in prolonged sitting (i.e., sitting bouts ≥ 30 min) (4).

Individuals who spend prolonged periods being sedentary have an increased risk of adverse health outcomes, including type 2 diabetes, cardiovascular disease, some cancers, and mental health problems such as depression and anxiety (5–9). This has led to incorporating a focus on reducing and breaking up sedentary behavior in national and international physical activity guidelines (10, 11). As high amounts of sitting are accumulated in the office workplace, this is a key setting where interventions could be implemented to improve occupational and public health. Indeed, an expert consensus statement recommended that workers who are in predominantly desk-based occupations should start by accumulating 2 h of standing and light activity during working hours each day, and progress to reaching a target of 4 h per day (12).

There is a growing body of evidence that suggests height-adjustable workstations (i.e., a workstation that permits the user to interchangeably work in a seated or standing posture) could be effective for decreasing sitting in the workplace (13). When height-adjustable workstations are provided as part of multi-component interventions that also include strategies such as education, manager support, standing meetings, self-monitoring and social support, workplace sitting has been significantly reduced by 45 to 83 min/work shift after 12 months (14, 15). Although these interventions appear promising, management of organizations might be reluctant to implement them if productivity is negatively affected (16). With respect to productivity concerns, a systematic review reported that productivity is likely to be unaffected by using a height-adjustable workstation (17). There are also limited studies that have reported taking employee's perceptions and experiences into account when developing interventions to reduce workplace sitting (18), which may limit intervention adherence and effectiveness. By involving the target audience in the development of interventions, this will help to ensure their needs are met and enhance their adherence and engagement (19). The Behavior Change Wheel (BCW) (20, 21) has been recommended for guiding the systematic process of developing an intervention using a framework that takes into account the needs of the target audience. To the author's knowledge, only one previous office workplace intervention has been developed using the BCW (18); the SMArT Work intervention in National Health Service employees led to significant reductions in sitting at work and improvements in job performance, work engagement, occupational fatigue, sickness presenteeism, anxiety and quality of life over 12 months (15). Further research is required in other occupational groups to strengthen the evidence base for incorporating a focus on reducing and breaking up sitting in workplace policy and practice. Demonstrating the effectiveness of such interventions for improving work-related outcomes, such as worker productivity, will be important for encouraging the adoption of such changes in policy and practice. The use of ecological momentary assessment (EMA) facilitated through smartphone technology provides the opportunity to collect simultaneous time-stamped data with limited recall bias to elucidate on the temporal effects of posture and activities on work performance throughout the workday (22, 23). However, the effects of workplace sitting interventions on EMA measures of work-related outcomes is limited.

This article describes the protocol for a pilot cluster-randomized controlled trial (RCT) to evaluate the feasibility and efficacy of a multi-component intervention to reduce and break up workplace sitting, including the systematic process for identifying the content of the intervention using the BCW. The effects of the intervention on a range of sitting, activity, health, wellbeing and work-related outcomes (including EMA assessment) will be evaluated.

## Methods

### Study Design and Overview

**Figure 1:**
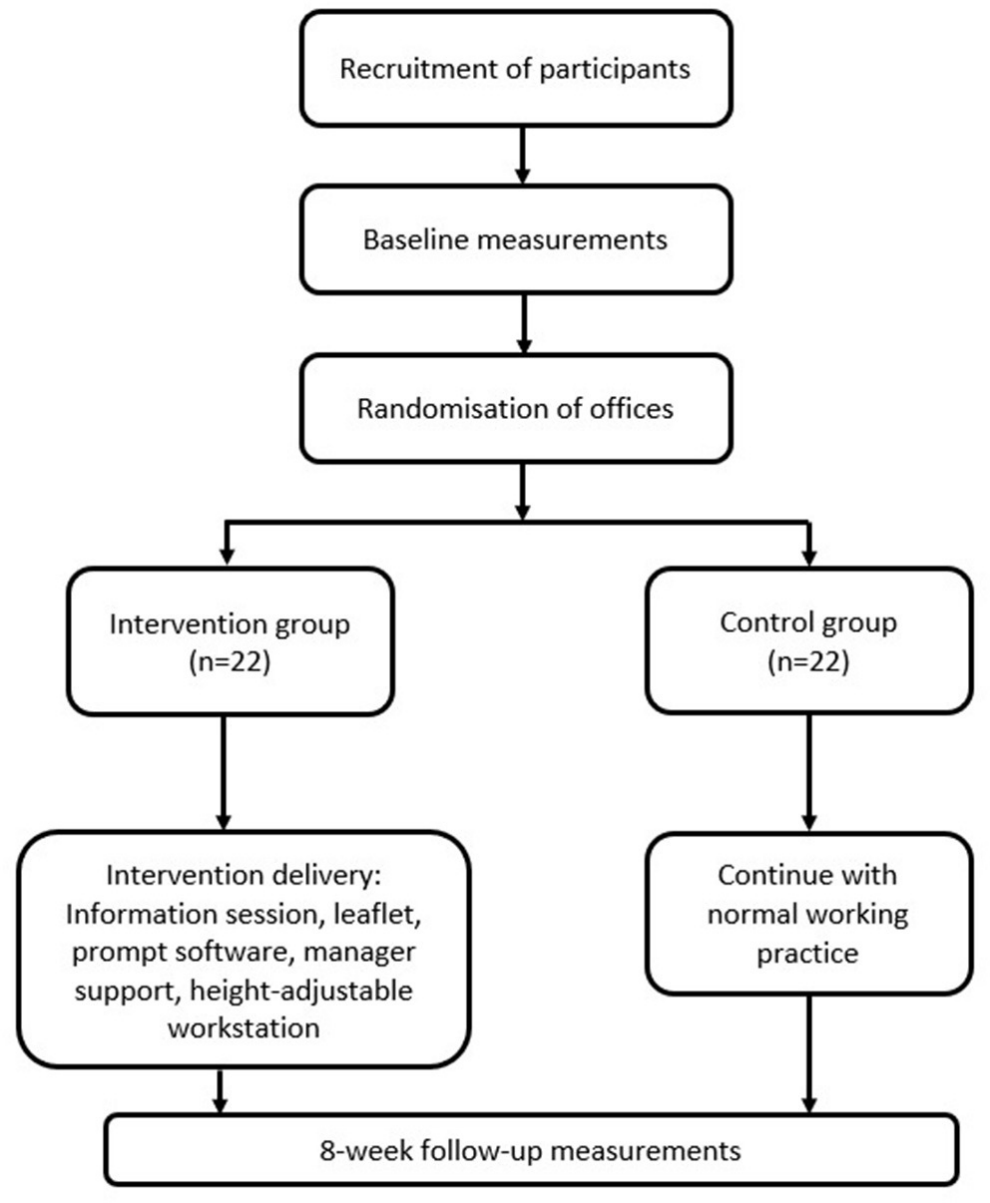
Overview of study design.

The study adopts an 8-week, two-arm cluster RCT design that is planned to be conducted during Spring and Summer months (see Figure 1 for overview of study design). The unit of randomization will be the workers' offices in line with previous research (24). Participants will be randomly allocated into clusters with each cluster being in either the control or intervention group for a period of 8 weeks. Given the nature of the intervention, blinding from participants or the core research team is not possible.

The study is registered on ClinicalTrials.gov (NCT03560544). Ethical approval for the procedures detailed below was obtained from the University of Bedfordshire Institute for Health Research Ethics Committee (lHREC836). Participants will provide written informed consent prior to any testing procedures and the study is being conducted in accordance with the Declaration of Helsinki.

### Study Setting

The study will be conducted with office workers employed across a university campus and a local council, both located in the East of England region, UK. Participants were recruited from one campus at the university and employees at the council worked within offices at a single site. Clusters of participants will be recruited from both organizations who will be randomly allocated to the intervention or control group i.e., there will be intervention and control clusters located at each organization.

### Participant Recruitment

Management approval will be sought and obtained for the recruitment of the employees, changes to the workplace environment, and for study testing and communications to take place during work time. The project will be advertised to staff as part of a workplace wellbeing programme via internal mail, staff meetings, information fliers in offices and other strategic places within the participating organizations.

### Eligibility Criteria

Participants will be full-time desk-based office employees between the ages of 18 and 60 years employed in any job type and from any ethnic background. Individuals will be excluded from the study if they are pregnant, have a history of musculoskeletal complaints, non-ambulatory, or have a planned holiday that would mean they would not be at work for more than 2 weeks during the 8-week intervention period.

### Sample Size

There is no standard procedure for determining the sample size for pilot studies as their main purpose is to obtain an estimate of the standard deviation that could be used to determine the likely effect size to inform a full trial (25). Thus, the proposed study will recruit a total of 44 participants in two groups of 18 allowing for an attrition rate of 20%. This is in line with sample size recommendations for pilot studies (26) and that used in a previous related pilot study of a workplace intervention to reduce sitting (27).

### Randomization

To minimize contamination between groups in open plan offices, a cluster randomization approach will be used with offices being the unit of randomization i.e., randomization into either the intervention or control group will be assigned on an office basis (28). The number of clusters is projected to be approximately eight with an average cluster size of six (29). Randomization will be achieved by assigning a cluster ID to each office and clusters then being allocated to intervention or control group using an online randomization tool (https://www.randomlists.com/team-generator) by an independent researcher (30). Randomization will take place before the collection of baseline measurements and group allocation will not be disclosed to participants until the completion of baseline assessment (28).

### Intervention Development, Content and Delivery

The intervention described in this study protocol was developed by systematically following the BCW framework (20, 31). The first step was to identify the problem (health and cardiovascular disease risk) in behavioral terms (sedentary behavior) and specify the target behavior to be changed (sitting at work). This was then followed by a COM-B (Capability, Opportunity and Motivation-Behavior) behavioral analysis conducted for the identification of barriers and facilitators to breaking up workplace sitting in office workers (32). The analysis led to the identification of seven core themes [“*Knowledge-deficit sitting behavior,” “Willingness to change,” “Inadequate cognitive resources for action,” “Tied to the desk,” “Competing motivations,” “Emotional influences,” and “Organizational support and interpersonal influences”* (32)], which linked to five of the six COM constructs and 12 domains of the Theoretical Domains Framework (TDF) (33). The TDF serves as a theoretical lens for understanding the cognitive, affective, social and environmental influences on behavior (33, 34). Subsequently, seven possible intervention functions, three policy categories and 38 behavior change techniques (BCTs) based on the BCT Taxonomy version 1 (35) that could be considered for inclusion in an intervention for reducing and breaking up workplace sitting were identified (36).

In deciding which intervention functions, policy categories and BCTs were most appropriate or have the highest possibility of success in delivering the desired change in this context, the Acceptability, Practicability, Effectiveness/cost-effectiveness, Affordability, Safety/side-effects, Equity (APEASE) criteria was applied. This is line with recommendations for using the BCW in practice (21, 37). Initial judgments using the APEASE criteria were made by the first author and reviewed by the co-authors (AC, DB and DH) until consensus regarding acceptance or rejection was reached. This led to identifying for inclusion, five intervention functions (Education, Persuasion, Enablement, Training and Environmental Restructuring; see Table 1), three policy categories (Communication/Marketing, Guidelines, and Environmental/Social Planning; Table 2) and 17 BCTs [*Goal setting (behavior), Action planning, Problem solving, Instruction on how to perform the behavior, Credible source, Information about health consequences, Information about emotional consequences, Prompts/cues, Social support [unspecified], Restructuring the physical environment, Restructuring the social environment, Adding objects to the environment, Behavioral practice/rehearsal, Behavior substitution, Habit formation, Habit reversal and Demonstration of the behavior*; see Table 3]. Table 4 shows a logic model, whereby the intervention content specified by BCTs is mapped onto components of the TDF and COM-B model. It also includes description of the delivery mode for the intervention content. The intervention thus includes a combination of education, persuasion, training, enablement, and environmental restructuring with the aim of addressing all of the identified constructs of the COM-B model and TDF. The intervention strategies and modes of delivery consist of an information session provided by a researcher and a leaflet covering the health consequences of prolonged sitting and the benefits of breaking up sitting, goal setting, action planning; computer prompt software; line manager support; and a height-adjustable workstation at each individual's desk. The computer prompt Google Chrome extension “Marinara: Pomodoro Assistant” will be installed on participant's computers. This prompt software has a customisable timer that can be used to set prompt alerts that appear as pop-up messages on the screen to remind the user to take a break. The break duration is customisable and the user is notified when the break has ended.

**Table 1:** Selection of intervention functions to cause a change in sitting behavior in desk-based employees.

**Intervention function**	**A**	**P**	**E**	**A**	**S**	**E**	**Comments**
Education	✓	✓	✓	✓	✓	✓	Information regarding the consequences of prolonged sitting and why sitting should be interrupted is readily available.
Enablement	✓	✓	✓	✓	✓	✓	Permission that permits participants to break up sitting is already granted by employee's management.
Training	✓	✓	✓	✓	✓	✓	Instruction on how to carry out the proposed sitting behavior will be provided and demonstrated to participants.
Persuasion	✓	✓	✓	✓	✓	✓	Safe resources for the delivery of this function are readily available. For instance, consequences of uninterrupted sitting, information regarding sitting guideline and participants can be supported in setting targets and planning to achieve them.
Incentivisation	✓					✓	No funds available, therefore it is not practicable or affordable. No evidence for effectiveness or side-effects.
Environmental restructuring	✓	✓	✓	✓	✓	✓	Made possible due to equipment donation.
Modeling	✓		✓	✓	✓	✓	Not practicable to identify a timekeeper who could get people moving as participants will be in clusters in different offices.

*(Left to right) A, Acceptability; P, Practicality; E, Effectiveness; A, Affordability; S, Side-effect/safety; E, Equity; ✓, Intervention function meets APEASE criterion*.

**Table 2:** Identification of policy categories to support the intervention delivery.

**Policy categories**	**A**	**P**	**E**	**A**	**S**	**E**	**Comments**
Communication /Marketing	✓	✓	✓	✓	✓	✓	These policies are relevant as there is a need to educate participants about what to do and why change is important.
Guidelines	✓	✓	✓	✓	✓	✓	Although development of new guidelines is not warranted, participants' attention will be drawn to existing guidelines for sitting among office workers.
Environmental/Social Planning	✓	✓	✓	✓	✓	✓	Permission will be obtained from the participating organization management to modify the offices.

*(Left to right) A, Acceptability; P, Practicality; E, Effectiveness; A, Affordability; S, Side-effect/safety; E, Equity; ✓, Policy category meets APEASE criterion*.

**Table 3:** Screening of the possible behavior change techniques (BCTs) with APEASE criteria.

**BCT (code) [Obtained from a preceding qualitative study (32)]**	**A**	**P**	**E**	**A**	**S**	**E**	**Comments**
Goal setting (behavior) (1.1)	✓	✓	✓	✓	✓	✓	
Problem solving (1.2)	✓	✓	✓	✓	✓	✓	
Action planning (1.4)	✓	✓	✓	✓	✓	✓	
Monitoring of behavior by others without feedback (2.1)	✓		✓	✓	✓	✓	Not practicable as colleagues will not have time.
Feedback on behavior (2.2)	✓		✓		✓	✓	Not affordable and practicable in this context, as a monitoring device needs be worn then retrieved before feedback can be provided.
Self-monitoring of behavior (2.3)	✓	✓	✓		✓	✓	Not affordable in this context as a monitoring device will need to be provided for and worn by all the participants.
Self-monitoring of outcome(s) of behavior (2.4)	✓		✓	✓	✓	✓	The intervention will be short-term. Therefore, it may be difficult and not practicable to measure and observe any noticeable change.
Social support (unspecified) (3.1)	✓	✓	✓	✓	✓	✓	
Social support (practical) (3.2)			✓	✓	✓	✓	Practical help from other colleagues does not appear acceptable or practical due to limited time and competing priorities of colleagues.
Instruction on how to perform the behavior (4.1)	✓	✓	✓	✓	✓	✓	
Information about Antecedents (4.2)	✓		✓	✓	✓	✓	Participants might feel overburdened by needing to record events taking place around sitting periods.
Behavioral experiments (4.4)	✓			✓	✓	✓	Although advice regarding consequences of prolonged siting behavior will be provided based on existing evidence, it will be difficult in terms of cost-effectiveness and practicality to collect data to advise the participants prior to the intervention.
Information about health consequences (5.1)	✓	✓	✓	✓	✓	✓	
Information about social and environmental consequences (5.3)	✓			✓	✓	✓	Not practicable as information is not known.
Information about emotional consequences (5.6)	✓	✓	✓	✓	✓	✓	
Demonstration of the behavior (6.1)	✓	✓	✓	✓	✓	✓	
Social comparison (6.2)		✓	✓	✓		✓	Revealing information about others' sitting time would breach confidentiality, which may be deemed unacceptable. Comparing to others may make others feel uncomfortable as a side effect.
Information about others' approval (6.3)	✓			✓	✓	✓	Not practicable as it is difficult to know what others think about sitting behavior in the workplace.
Prompts/cues (7.1)	✓	✓	✓	✓	✓	✓	
Behavioral practice/rehearsal (8.1)	✓	✓	✓	✓	✓	✓	
Behavior substitution (8.2)	✓	✓	✓	✓	✓	✓	
Habit formation (8.3)	✓	✓	✓	✓	✓	✓	
Habit reversal (8.4)	✓	✓	✓	✓	✓	✓	
Credible source (9.1)	✓	✓	✓	✓	✓	✓	
Material incentive (behavior) (10.1)	✓		✓		✓	✓	Not practicable, affordable or sustainable in this context.
Material reward (behavior) (10.2)	✓		✓		✓	✓	Not practicable, affordable or sustainable in this context.
Non-specific reward (10.3)	✓		✓		✓	✓	Not practicable, affordable or sustainable in this context.
Social reward (10.4)	✓		✓	✓	✓	✓	Not practicable (time consuming).
Social incentive (10.5)	✓		✓	✓	✓	✓	Not practicable (time consuming).
Non-specific incentive (10.6)	✓		✓		✓	✓	Not practicable, affordable or sustainable in this context.
Incentive (outcome) (10.8)	✓		✓		✓	✓	Not practicable, affordable or sustainable in this context.
Self-reward (10.9)	✓		✓		✓	✓	Not practicable or affordable as participants may not have time or ability to self-praise or have money to buy themselves gifts as a reward for breaking up sitting.
Reduce negative emotions (11.2)	✓		✓	✓	✓	✓	Not practicable as would require time and support from others to provide personalized advice.
Restructuring the physical environment (12.1)	✓	✓	✓	✓	✓	✓	
Restructuring the social environment (12.2)	✓	✓	✓	✓	✓	✓	
Adding object to the environment (12.5)	✓	✓	✓	✓	✓	✓	
Verbal persuasion about capability (15.1)	✓		✓	✓	✓	✓	Participants do not need any special skill to break up their sitting, however, there may be unseen physical limitations. So as not to pry into participants' privacy, it is considered not practicable.
Mental rehearsal of successful performance (15.2)	✓				✓	✓	Not practicable as it is difficult to ascertain if participants will have time to start imagining their ability to break up sitting while at work. It might be seen as a distraction and hence cannot be said to be practical and/or affordable. There is no data to confirm whether it could be effective.

*(Left to Right) A, Acceptability; P, Practicality; E, Effectiveness; A, Affordability; S, Side-effect/safety; E, Equity; ✓, BCT meets APEASE criterion*.

**Table 4:** Intervention content specified by behavior change techniques mapped onto components of TDF and COM-B model.

**Behavior change techniques**	**BCT definition (35)**	**COM-B construct targeted**	**TDF-domain targeted**	**Intervention function**	**Policy category**	**Intervention content and mode of delivery**
1.1 Goal setting (behavior)	Set or agree on a goal defined in terms of the behavior to be achieved	Reflective motivation	Goals	Enablement	Communication/Marketing	Participants will be advised and supported face-to-face to set bi-weekly goals for the duration of time spent sitting. This means that they will set different target for weeks 2, 4 and 6 to achieve both the minimum (2 h) and maximum (4 h) time for standing at work as advised in an expert statement (12).
1.2 Problem solving	Analyse, or prompt the person to analyse, factors influencing the behavior and generate or select strategies that include overcoming barriers and/or increasing facilitators	Psychological capability; Reflective motivation	Behavioral regulation; Goals	Enablement	Communication/Marketing	Ideas for problem solving will be provided in an information leaflet and within emails sent by managers providing support for engaging in the intervention. The research team will support participants in developing an action plan in relation to how they will break up their sitting to achieve sitting/standing targets (e.g., to stand every 30 min during the working day) and how to manage this around competing work duties. This will include how they will use computer prompt software to support the behavior. Computer prompt software will be installed on participants' computers to support frequent alternations in posture in addition to bi-weekly emails from line managers to encourage participants to work toward their targets.
1.4 Action planning	Prompt detailed planning of performance of the behavior (must include at least one of context, frequency, duration and intensity).					
3.1 Social support (unspecified)	Advise on, arrange or provide social support (e.g., from friends, relatives, colleagues, buddies or staff) or non-contingent praise or reward for performance of the behavior	Social opportunity; Physical opportunity; Reflective motivation	Social influences; Environmental context and resources; Social/Professional role and identity	Enablement; Environmental restructuring	Environmental/Social planning;	Approval will be sought and obtained from participating organization management. Participants will be assured via email from managers that they will not be penalized by participating in the study. Line managers will send bi-weekly emails that contain tips for breaking up sitting, appreciating commitment of the participants working toward breaking up prolonged sitting and reminding them of their bi-weekly goals in weeks 2, 4 and 6 (24). The programme will be designed in such a way that at least two people from the same office will participate in the intervention. This will provide the participants the opportunity to support one another and reduce the feeling of aloneness or being judged.
4.1 Instruction on how to perform the behavior	Advise or agree on how to perform the behavior (includes “Skills training”) Note: when the person attends classes	Psychological capability	Skills; Knowledge	Training; Education	Guidelines	A face-to-face training session will be provided to the participants by one member of the research team on how to break up sitting using prompt software and a height-adjustable workstation. Definition of prolonged sitting (sitting > 30 min) will be provided and participants will be advised to not sit more than 30 min in one continuous bout (28, 38). Participants will be provided with a leaflet containing information on the recommended sitting time guidelines, which states that office workers should be on their feet at work, for a minimum of 2 h initially and progressively increase to 4 h per day.
5.1 Information about health consequences	Provide information (e.g., written, verbal, visual) about health consequences of performing the behavior	Psychological capability; Reflective motivation	Knowledge; Belief about consequences	Education; Persuasion	Communication/Marketing; Guidelines	A face-to-face information session will be organized, and participants will be educated about information regarding the health consequences of prolonged sitting and the benefits of breaking up sitting. A leaflet containing the information will also be provided.
5.6 Information about emotional consequences	Provide information (e.g., written, verbal, visual) about emotional consequences of performing the behavior	Psychological capability; Reflective motivation	Knowledge; Belief about consequences	Education; Persuasion	Communication/Marketing	Verbal information regarding some of the emotional consequences (such as anxiety and depression) of prolonged sitting will be provided during a 10-min information session.
6.1 Demonstration of the behavior	Provide an observable sample of the performance of the behavior, directly in person or indirectly	Psychological capability	Skills; Knowledge	Training; Education; Modeling; Persuasion	Guidelines, Communication/Marketing	A step-by-step guide on how the intervention is being delivered will be provided to participants in a leaflet and a demonstration of intervention components will be delivered to participants (27).
7.1 Prompts/cues	Introduce or define environmental or social stimulus with the purpose of prompting or cueing the behavior.	Physical opportunity; Psychological capability	Environmental context and resources; Memory, attention and decision processes	Environmental restructuring; Enablement	Environmental/Social planning	Prompt software will be installed on participants' computers. This software will be used to provide reminders to change between sitting and standing, and to remind participants to take short breaks (e.g., walking) based on participant preference.
8.1 Behavioral practice/rehearsal	Prompt practice or rehearsal of the performance of the behavior one or more times in a context or at a time when the performance may not be necessary, in order to increase habit and skill	Physical opportunity	Environmental context and resources; Skills	Environmental restructuring; Training	Environmental/Social planning	A Work-Fit-T Sit-Stand Desktop Workstation (Ergotron, St Paul, MN, USA) will be installed at participants' regular desks. This will provide them with the opportunity to alternate between sitting and standing while working.
8.2 Behavior substitution	Prompt substitution of the unwanted behavior with a wanted or neutral behavior	Physical opportunity; Psychological capability	Environmental context and resources; Memory, attention and decision processes	Environmental restructuring; Enablement	Environmental/Social planning	Installation of a Work-Fit-T Sit-Stand Desktop Workstation (Ergotron, St Paul, MN, USA) and prompt software on participant's computers will be used to provide reminders for participants to alternate between standing and sitting and form new habits around sitting less. Participants will be encouraged within the information leaflet and meeting with a researcher to intermittently substitute sitting with standing work
8.3 Habit formation	Prompt rehearsal and repetition of the behavior in the same context repeatedly so that the context elicits the behavior					
8.4 Habit reversal	Prompt rehearsal and repetition of an alternative behavior to replace an unwanted habitual behavior					
9.1 Credible source	Present verbal or visual communication from a credible source in favor of or against the behavior	Psychological capability; Reflective motivation	Knowledge; Beliefs about consequences	Education; Persuasion	Communication/Marketing	A verbal communication based on an expert statement (12) about the need for office workers to reduce sitting and how this could be achieved will be provided by the researchers. The information will also be available in a leaflet.
12.1 Restructuring the physical environment	Change, or advise to change the physical environment in order to facilitate performance of the wanted behavior or create barriers to the unwanted behavior	Physical opportunity	Environmental context and resources	Environmental restructuring	Environmental/Social planning	Height-adjustable workstations will be installed on participant's regular desks to afford them the opportunity to alternate between sitting and standing while working.
12.5 Adding object to the environment	Add objects to the environment in order to facilitate performance of the behavior					
12.2 Restructuring the social environment	Change, or advise to change the social environment in order to facilitate performance of the wanted behavior or create barriers to the unwanted behaviur (other than prompts/cues, rewards and punishments)	Social opportunity	Social influences; Environmental context and resources	Enablement; Environmental restructuring	Environmental/Social planning	Intervention participants will be cluster randomized so that others in their office are taking part, which will help prevent feelings of being isolated or criticized and promote peer support.

*TDF, Transtheoretical Domains Framework; COM-B, Capability Opportunity and Motivation-Behavior; BCT, behavior change technique*.

### Control Group

The control group will continue to work as normal with no changes in their routine or environment. They will complete the same measurements as the intervention group.

### Measurements

All measures for both intervention and control groups will be performed at baseline and during the final week of the 8-week study period. Questionnaires will be completed online using Qualtrics survey software (Qualtrics, Provo, Utah, USA) with physical measures taking place at participant's workplaces in a private room.

#### Primary Outcome Measure: Workplace Sitting Time

The primary outcome measure is sitting time at work, which will be measured using an activPAL3 activity monitor (PAL Technologies Ltd., Glasgow, UK). The activPAL is a portable lightweight device, which is usually worn on the front of the mid-thigh to take measurement of postural information by recognizing periods of lying down/sitting, standing, and stepping based on the inclination of the thigh (39, 40). The activPAL has high reliability and validity for assessing sitting, standing and stepping (41). In this study, the device will be worn on the right thigh for seven full consecutive days (42) at baseline and during the last week of the intervention. The device will be waterproofed by wrapping it within a nitrile sleeve and an adhesive medical dressing. In addition, participants will complete a diary to keep record of their waking time, working hours and time they go to bed while wearing the device to enable calculation of waking and working time wear periods (42). Data from the activPAL will be considered valid only if the device is worn for a minimum of 75% of the workplace monitoring time (39, 40). The sitting time at work variable will be normalized by expressing it as a percentage of the duration of the working day for each participant.

#### Secondary Outcome Measures

##### Sitting, Standing and Stepping

In addition to workplace sitting, the activPAL will be used to measure total daily sitting time and the following variables for working hours and across the waking day: time spent in sitting bouts <30 min and ≥30 min, the number of sitting bouts <30 min and ≥30 min, standing time, number of sit-to-upright transitions, number steps and stepping time. Processing PAL (v1.1, University of Leicester, Leicester, UK) will be used to process the data. A validated algorithm (43) will be used to identify valid waking wear data with heat maps being generated to visually check classifications of waking wear time against information provided in the participant's diaries. Waking bout corrections will be made where errors are identified (42). A valid day for daily data will be accepted if wear time is ≥10 h per day; ≥500 steps per day and if not recording > 95% data in one activity category (i.e., sitting, standing or stepping). The resulting data for all primary and secondary activPAL outcome measures will be averaged across all valid days. All time variables for the working day will be normalized by expressing them as a percentage of the work shift duration. All count variables, such as the number of sitting bouts, will be normalized by expressing them as counts per hour.

##### Adherence Analysis

Adherence to the intervention will be assessed using activPAL data for the working day. Participants will be classified into one of three groups at baseline and follow-up in line with recommendations from an expert statement on reducing sedentary time in the workplace (12): (1) meeting the recommended guidelines (MEETING) for standing and/or stepping at work of ≥4 h per work day, (2) meeting the minimal guidelines (MINIMAL) for standing and/or stepping at work of ≥2 h but <4 h per work day, or (3) sedentary (SED) i.e., not meeting MINIMAL or MEETING guidelines.

##### Feasibility

Feasibility will be assessed based on recruitment response rate (percentage of people who express interest in participating in the study / number of invitations sent out to individuals x 100) and eligibility rate (percentage of individuals who are screened / number of individuals eligible to take part x 100). Retention rate will be calculated as number of participants who complete 8-week measures / number of participants enrolled into the study x 100. Data completion rates will be calculated as the number of complete datasets for each outcome measure / number of participants enrolled ×100.

##### Intervention Acceptability

Acceptability of the intervention will be assessed using semi-structured interviews with intervention participants. Data will be analyzed using thematic analysis as well as deductive charting using the APEASE (Acceptability, Practicality, Effectiveness, Affordability, Side effects, Equity) criteria (44).

##### Productivity

Work productivity will be assessed using measures of absenteeism, presenteeism, and ecological momentary assessment (EMA) at baseline and during week eight of the study. Absenteeism will be measured using self-report at baseline and at the end of the eighth week. Three questions to assess unsanctioned absences will be used from a previous study (45). This includes the number of days off work in the last 2 weeks for sickness (colds, flu, etc.), the number of days off work for mental health reasons (stress, burnout, etc.), and the number of days excused work (e.g., compassionate leave, educational leave, parental leave). Self-reported absenteeism will be calculated by summing the number of days reported across all three questions. Presenteeism will be measured using the 8-item Work Limitations Questionnaire (WLQ), which is a short version of the original 25-item WLQ (46). This version of the WLQ is a reliable and valid alternative to the longer questionnaire (47). The WLQ requires participants to self-report their level of difficulty (or ability) to perform eight specific job demands in the last 2 weeks, with answers grouped into four work limitation scales on time management, physical, mental, and output demands.

The final measure of productivity is EMA, which has been used previously in a variety of different contexts to measure physical activity and sedentary behavior (48–50). Prompt software will be installed on participant's smartphones to deliver the EMA. Prompts to complete the EMA will occur at random, four times each week day between 9 am to 5 pm (51). In total, participants will be requested to respond to and complete a maximum of 12 EMA surveys in a week. Once a participant has completed 12 surveys, the prompt software will no longer prompt that participant to respond (51). The questionnaire includes items about posture, musculoskeletal issues, task being performed, social interactions, mood, and perception of engagement and productivity (Table 5), typically taking <1 min to complete each time.

**Table 5:** Ecological momentary assessment productivity questions.

**Survey question**	**Responses**
What posture are you in?	Sitting/Standing/Walking
Are you experiencing musculoskeletal pain right now?	Yes/No
What are you currently doing?	In a meeting/Working at my desk/Working away from my desk/Eating/In transit/On a break/Other
How many people are you doing this with?	Alone / With 1 other person/ With 2–5 people/With 6+ people
On a scale of 1 to 10, 1 being not at all and 10 being extremely, please rate your emotions at this moment	
Happy	
Stressed	
Energized	
Anxious	
Productive	
Motivated	
Engaged	
Creative	

##### Cardiometabolic Risk Biomarkers

All cardiometabolic risk biomarker measures will be taken at baseline and within 5 days post-intervention. This includes the following:

###### Fasting Blood Glucose and Lipid Profile.

Blood samples taken after an overnight fast of at least 10 h using a finger prick method with a lancet (52). Blood samples will be analyzed with a Cholestech LDX analyzer (Cholestech Corp., Hayward, CA., USA) to provide measures of total cholesterol, high-density lipoprotein cholesterol, low-density lipoprotein cholesterol, triglycerides and blood glucose (52).

*Blood Pressure*. Resting systolic and diastolic blood pressure will be measured on the right arm after resting for at least 10 min in a seated position using an automated blood pressure monitor (Omron HEM705 CP, Omron Healthcare UK Limited, Milton Keynes, UK) validated by the European Society of Hypertension (53). The average of two blood pressure readings with a 2-min rest between each will be used for analysis.

*Anthropometry*. The standardized procedure of Lohman et al., (54) will be used. Height is measured with a stadiometer (Horltain Ltd., Crymych, UK) to the nearest 0.1 cm, while mass is measured with an electronic weighing scale (Tanita Corp., Tokyo, Japan) to the nearest 0.1 kg. Waist circumference is recorded to the nearest 0.1 cm measured around the waist at navel-level while participants are standing (55, 56). All measures will be taken by the same investigator to avoid inter-investigator variability. Body mass index will be calculated as: weight / height^2^.

##### Psychological and Mental Wellbeing

Perceived levels of stress will be measured using the Cohen Perceived Stress Scale (57), which evaluates the unpredictability, uncontrollability and overload of an individual's life, and has high validity and reliability (57). The questionnaire requires participants to rank the frequency of feelings and thoughts using a five-point Likert scale ranging from 1 “*Never*” to 5 “*Very often”*. Participant's mood states will be measured using the Positive and Negative Affect Schedule (PANAS), which is a 20-item self-report measure of positive affect and negative affect (58). The questionnaire makes use of words that describe feelings and emotions on a scale from 1 “*Very slightly or Not at all”* to 5 “*Extremely*.” The PANAS positive and negative affect scales have high reliability (59). Changes in participants' mental wellbeing will be assessed using the Warwick-Edinburgh Mental Well-Being Scale (WEMWBS) (60), which is a 14-item scale that explores feelings and thoughts over the last 2 weeks. This study was registered to obtain permission to use WEMWBS (ID: 448654978) on warwick.ac.uk. The WEMWBS classifies individuals as having low, average or high mental wellbeing and can measure changes over time. The questionnaire asks questions such as “I have been feeling confident” and participants will need to rate themselves on a scale from 1 “*None of the time”* to 5 “*All of the time.”* It has been reported to have high test-retest reliability (61).

### Statistical Analysis

All statistical analyses will be performed using SPSS (IBM Corp, Armonk, New York, USA). Data will be assessed for normality using the Shapiro-Wilk test owing to the relatively small sample size. Linear mixed models will be used with arm and time as fixed effects, participant ID and cluster ID as random effects, and the baseline value of each variable as a covariate. An additional linear mixed model will be used to compare the effect of standing vs. sitting posture for the EMA variables at follow-up with posture and arm as fixed effects, participant ID and cluster ID as random effects, and the baseline value of each variable as a covariate. The Sidak *post-hoc* adjustment will be used to adjust for multiple comparisons in all models. Adjustments for missing data will be made using the estimated marginal means procedure of the linear mixed models. If there was a large number of non-normally distributed variables, the bias-corrected and accelerated (BCa) bootstrap method will be used to produce unbiased estimates of the confidence limits for all data (62). Data will be presented as mean and 95% confidence intervals of the bootstrapping procedure. The level of statistical significance will be *p* ≤ 0.05 for two-tailed tests. Magnitude of effects will be reported as Hedges' *g*, which is a corrected effect size based on Cohen's *d* that is suitable for use in small sample sizes of 20 or less per group to produce an unbiased estimate of the population effect size (63). The magnitude of the effect sizes will be considered to be small if they are ≥ 0.2, moderate if ≥ 0.6, and large if ≥ 1.2, using the scale proposed by Hopkins et al. (64). Adherence to the intervention will be assessed using the Wilcoxon signed rank test to compare baseline and follow-up classifications. The standardized test statistic of the Wilcoxon test will be converted to an effect statistic by dividing it by the square root of N (65), which can then be interpreted as *r* using the scales of Cohen (66) and Hopkins et al. (64).

## Discussion

This pilot cluster-randomized controlled trial will evaluate the efficacy of a tailored workplace intervention for reducing and breaking up sitting, productivity, wellbeing and cardiometabolic risk biomarkers. The multi-component intervention protocol was designed using a participatory approach informed by the BCW and identification of intervention functions, policy categories and BCTs using the APEASE criteria to enhance the intervention's adherence and effectiveness. In addition to employing this systematic and evidence-based process for developing the intervention, further strengths of this study include the cluster-randomized design to minimize contamination between groups that are located within the same organization. The study will also be conducted within an ecologically valid office environment, thus enhancing its real-world application. Ecological momentary assessment will be used to assess temporal effects of the intervention on productivity during specific postures and work activities; this may provide important evidence to encourage changes in workplace policy and practice. Potential limitations include sample representativeness of the general office worker population as the study is being conducted only within two organizations. It will also not be possible to determine the effects of each individual strategy used as part of the intervention, which could help in determining their suitability for inclusion in occupational health promotion programmes. Furthermore, physical activity levels prior to taking blood samples will not be standardized. This may be a limitation as there is the potential for carryover effects of prior exercise on glucose and lipid levels.

In conclusion, this study will provide important evidence regarding the efficacy of a systematically developed tailored workplace intervention. This will include evaluating the effects of the intervention on a range of health and work-related outcomes, including an ecological momentary assessment of productivity that has been seldom employed in office workplace studies; this may be key in promoting the adoption and implementation of such interventions in workplace practice and policies. The findings of this study can be used to inform a future fully powered RCT to determine the effectiveness of this multi-component intervention for reducing sitting and improving health in office workers.

## Ethics Statement

The studies involving human participants were reviewed and approved by the University of Bedfordshire Institute for Health Research Ethics Committee. The patients/participants provided their written informed consent to participate in this study.

## Author Contributions

SO contributed to the concept, development of intervention, and drafting the manuscript. DB, DH, and AC contributed to the concept, development of intervention, and manuscript review. All authors reviewed and approved the final version of the manuscript.

## Conflict of Interest

The authors declare that the research was conducted in the absence of any commercial or financial relationships that could be construed as a potential conflict of interest.

## Publisher's Note

All claims expressed in this article are solely those of the authors and do not necessarily represent those of their affiliated organizations, or those of the publisher, the editors and the reviewers. Any product that may be evaluated in this article, or claim that may be made by its manufacturer, is not guaranteed or endorsed by the publisher.
